# Establishment of papillary thyroid cancer organoid lines from clinical specimens

**DOI:** 10.3389/fendo.2023.1140888

**Published:** 2023-03-13

**Authors:** Hao Yang, Qingzhuang Liang, Jian Zhang, Jinkun Liu, Hao Wei, Haibo Chen, Wei Wei, Dong Chen, Yongsheng Zhao

**Affiliations:** ^1^ Department of Nuclear Medicine, Peking University Shenzhen Hospital, Shenzhen, Guangdong, China; ^2^ Department of Thyroid and Breast Surgery, Peking University Shenzhen Hospital, Shenzhen, Guangdong, China; ^3^ Department of Thyroid and Breast Surgery, Shenzhen Luohu Hospital Group Luohu People’s Hospital, Shenzhen, Guangdong, China

**Keywords:** papillary thyroid cancer, organoid, 3D culture, clinical specimens, histological characterization, gene expression, preclinical model

## Abstract

Papillary thyroid cancer (PTC) is a common malignancy of the endocrine system, and its morbidity and mortality are increasing year by year. Traditional two-dimensional culture of cell lines lacks tissue structure and is difficult to reflect the heterogeneity of tumors. The construction of mouse models is inefficient and time-consuming, which is difficult to be applied to individualized treatment on a large scale. Clinically relevant models that recapitulate the biology of their corresponding parental tumors are urgently needed. Based on clinical specimens of PTC, we have successfully established patient-derived organoids by exploring and optimizing the organoid culture system. These organoids have been cultured stably for more than 5 passages and successfully cryopreserved and retried. Histopathological and genome analysis revealed a high consistency of the histological architectures as well as mutational landscapes between the matched tumors and organoids. Here, we present a fully detailed method to derive PTC organoids from clinical specimens. Using this approach, we have developed PTC organoid lines from thyroid cancer samples with a success rate of 77.6% (38/49) until now.

## Introduction

Thyroid cancer has become one of the most prevalent endocrine malignancies, with a rising global incidence in recent years (1). Thyroid cancer can be stratified into 4 pathological types: papillary thyroid cancer (PTC), follicular thyroid cancer (FTC), anaplastic thyroid cancer (ATC), and medullary thyroid cancer (MTC) (2, 3). Among different histologic types of thyroid cancer, PTC is the most common form, accounting for 80% to 85% of all cases (3–5). Thyroidectomy with radioiodine therapy is the first-line treatment mode for thyroid cancer, which can cure the majority patients with PTC. However, a subset of patients developed regional recurrence and/or distant metastasis, leading to an ineffective radioactive iodine treatment (6–8). In recent years, targeted therapy, especially small molecules targeting the receptor tyrosine kinases (RTKs) and BRAFV600E, has been evaluated in clinical trials with great promise for patients with progressive, radioactive iodine-refractory PTC (9–13). However, there is no suitable preclinical model to predict the therapeutic effect. Clinically relevant *in vitro* model that can faithfully represent PTC tissue is urgently needed.

For a long time, the majority of thyroid cancer experiments are based on mouse models and two-dimensional (2D) cultured cell lines (14). However, the two models have many disadvantages in clinical use. Cancer cell lines lack three-dimensional (3D) tissue architecture and have difficulty expressing the heterogeneity of the original tumor (15). Although the xenograft mouse models retain the genetic and histological features of patients’ tumor tissues, they are high technique required, time-consuming and expensive, which are difficult to be applied on a large scale (16). There has been an increased interest in organoids derived from healthy and diseased human tissues in the past several years. Formed by the induced pluripotent stem cells or organ progenitor cells, organoids are 3D cell complexes that are structurally and functionally similar to original organs or tissues. They have stable phenotype and genotype, and can be cultured for a long time *in vitro* (17). Many studies have shown that tumor organoids can highly recapitulate the characteristics of original tumor in morphology, pathology, genomic profiles, expression features and other aspects (18, 19).

Previously, we have successfully established PTC organoids and demonstrated that these models can retain the histological and genetic features of parental tumors (20). In this work, we provide a fully detailed, step-by-step protocol for the establishment of patient-derived PTC organoids from clinical samples. Using this method, we have developed PTC organoid lines from thyroid cancer specimens with a success rate of 77.6% (38/49).

## Key resources and materials

### Reagents

#### Peptides, proteins, and chemicals

Advanced DMEM/F12 (Gibco, USA, #12634-010)

Fetal bovine serum (FBS) (Gibco, #10091148)

HEPES (Gibco, USA, #15630-080)

GlutaMAX supplement (Gibco, USA, #35050-061)

Antibiotic-antimycotic (100×) (Gibco, USA, #15240-062)

B-27 supplement (50×), serum free (Gibco, USA, #17504-044)

N-acetyl-L-cysteine (Sigma-Aldrich, USA, #A9165; CAS: 616-91-1)

Nicotinamide (Sigma-Aldrich, USA, #N0636; CAS: 98-92-0)

SB202190 (Sigma-Aldrich, USA, #S7076; CAS: 350228-36-3)

A83-01 (Sigma-Aldrich, USA, #SML0788; CAS: 909910-43-6)

Recombinant human R-spondin 1 protein (Peprotech, USA, #120-38)

Recombinant human Noggin (Peprotech, USA, #120-10C)

Recombinant human EGF (Peprotech, USA, #AF-100-15)

Recombinant human FGF-7 (Peprotech, USA, #100-19)

Recombinant human FGF-10 (Peprotech, USA, #100-26)

Y-27632 dihydrochloride (Abmole, USA, #M1817; CAS: 129830-38-2)

DNase I (Roche Diagnostics, Switzerland, #11284932001)

Collagenase, type II (Gibco, USA, #17101015)

TrypLE Express (Gibco, USA, #12605010)

Recovery cell culture freezing medium (Gibco, USA, #12648010)

PBS (Gibco, USA, #10010023)

Matrigel Matrix Basement Membrane, growth factor reduced, Phenol Red-free (Corning, USA, #356231)

4’,6-diamidino-2-phenylindole (DAPI) (Beyotime, China, #C1002)

Antifade polyvinylpyrrolidone mounting medium (Sigma-Aldrich, USA, #10981)

10% neutral-buffered formalin (Solarbio, China, #G2161)

Agarose (Sigma-Aldrich, USA, #A9414)

Hematoxylin solution (Baso Diagnostics, China, #BA-4041)

Eosin solution (Baso Diagnostics, China, #BA-4099)

Ready-to-use normal goat serum (BOSTER, China, #AR0009)

Antigen retrieval buffer (Maixin Biotech, China, #MVS-0099)

TRIzol reagent (Invitrogen, USA, #15596026)

DNase I (Invitrogen, USA, #18047019)

RevertAid First Strand cDNA Synthesis Kit (Thermo Scientific, USA, #K1622)

Bestar^®^ Sybr Green qPCR Master Mix (DBI^®^ Bioscience, Germany, #DBI-2043)

### Antibodies

Galectin-1 (Gal-1) (1:300 dilution; Affinity Biosciences, China, #DF2383; RRID: AB_2839591)

Galectin-3 (Gal-3) (1:500 dilution; Abcam, USA, #ab76245; Clone: EP2775Y)

Cytokeratin 19 (CK19) (1:200 dilution; Maixin Biotech, China, #Kit-0030; Clone: A53-B/A2.26)

Ki-67 (1:200 dilution; Abcam, USA, #ab16667; Clone: SP6)

Cy3-labeled goat anti-rabbit IgG (H+L) (1:300 dilution; Beyotime, China, #A0516)

Cy3-labeled goat anti-mouse IgG (H+L) (1:300 dilution; Beyotime, China, #A0521)

### Materials

Biological samples (fresh): Human PTC tissue samples from surgery in Peking University Shenzhen Hospital, China

6-well clear TC-treated multiple well plates (Corning, USA, #3516)

0.22-µm filter (Millipore, USA, #SLGV033RB)

70-µm cell strainer (Corning, USA, #352350)

3 mL pasteur pipet (SORFA, China, #320411)

5 mL pasteur pipet (SORFA, China, #320511)

1.5 mL centrifuge tubes (biosharp, China, #BS-15-M-S)

15 mL centrifuge tubes (NEST Biotechnology, China, #601002)

50 mL centrifuge tubes (NEST Biotechnology, China, #602002)

10 mL serological pipet (Corning, USA, #357551)

Cryogenic vials (Thermo Fisher, USA, #375418)

Embedding cassettes (Beyotime, China, #FSR902)

Anti-off slides (Citotest Scientific, China, #188105)

Coverslips (Citotest Scientific, China, #10212440C)

### Solutions

1. AdDMEM/F12^+++^ (1×): 485 mL of advanced DMEM/F12 supplemented with: 5 mL antibiotic-antimycotic (100×), 5 mL GlutaMAX supplement (100×), and 5 mL HEPES (1M).

*Recommended storage time: at 4°C until the expiration date.*

2. Collagenase type II (250 mg/mL, 50×): 4 mL of 1 × AdDMEM/F12^+++^ supplemented with 1 g collagenase type II. Vortex and filter the solution with a 0.22-µm filter. Aliquot 100 µL into 1.5 mL microcentrifuge tubes for standby.

*Recommended storage time: at -20°C for up to 1 year.*

3. DNase I (10 mg/mL, 100×): 5 mL of 0.15 M NaCl supplemented with 50 mg DNase I. Filter the solution with a 0.22-µm filter. Aliquot 50 µL into 1.5 mL microcentrifuge tubes for standby.

*Recommended storage time: at -20°C for up to 6 months.*

4. Matrigel: Put Matrigel and new 1.5 mL microcentrifuge tubes on ice to cold down. Use cold sterile PBS to pre-cold P1000 tips. Aliquot required volume of Matrigel into 1.5 mL microcentrifuge tubes for standby.

*Recommended storage time: at -20°C until the expiration date.*

**NOTE:** Matrigel is rich in extracellular matrix proteins and basement membrane proteins. Repeated freezing and thawing should be avoided.

5. N-acetyl-L-cysteine (500 mM, 400×): Total volume of 20 mL ultra-pure water supplemented with 1.63 g N-acetyl-L-cysteine. Filter the solution with a 0.22-µm filter. Aliquot 125 µL into 1.5 mL microcentrifuge tubes for standby.

*Recommended storage time: at -20°C for up to 3 months.*

6. Nicotinamide (1 M, 100×): Total volume of 20 mL sterile PBS supplemented with 2.44 g Nicotinamide. Filter the solution with a 0.22-µm filter. Aliquot 500 µL into 1.5 mL microcentrifuge tubes for standby.

*Recommended storage time: at -20°C for up to 3 months.*

7. R-spondin 1 (100 µg/mL, 200×): 2.5 mL of sterile PBS supplemented with: 250 µg R-spondin 1, 0.1% (wt/vol) bovine serum albumin (BSA). Aliquot 250 µL into 1.5 mL microcentrifuge tubes for standby.

*Recommended storage time: at -20°C for up to 1 month.*

8. Noggin (100 µg/mL, 1,000×): 1 mL of sterile PBS supplemented with: 100 µg Noggin, 0.1% (wt/vol) BSA. Aliquot 50 µL into 1.5 mL microcentrifuge tubes for standby.

*Recommended storage time: at -20°C for up to 2 months.*

9. EGF (100 µg/mL, 2,000×): 1 mL of sterile PBS supplemented with: 100 µg EGF, 0.1% (wt/vol) BSA. Aliquot 25 µL into 1.5 mL microcentrifuge tubes for standby.

*Recommended storage time: at -20°C for up to 3 months.*

10. FGF-7 (100 µg/mL, 20,000×): 1 mL of sterile PBS supplemented with: 100 µg FGF-7, 0.1% (wt/vol) BSA. Aliquot 2.5 µL into 1.5 mL microcentrifuge tubes for standby.

*Recommended storage time: at -20°C for up to 3 months.*

11. FGF-10 (100 µg/mL, 10,000×): 1 mL of sterile PBS supplemented with: 100 µg FGF-10, 0.1% (wt/vol) BSA. Aliquot 5 µL into 1.5 mL microcentrifuge tubes for standby.

*Recommended storage time: at -20°C for up to 3 months.*

12. SB202190 monohydrochloride hydrate (30 mM, 3,000×): 0.453 mL of dimethyl sulfoxide (DMSO) supplemented with 5 mg SB202190 monohydrochloride hydrate. Aliquot 16.7 µL into 1.5 mL black microcentrifuge tubes for standby.

*Recommended storage time:* ***Keep in the dark*** *at -20°C for up to 3 months.*

13. A83-01 (25 mM, 50,000×): 0.474 mL of DMSO supplemented with 5 mg A83-01. Aliquot 1 µL into 1.5 mL microcentrifuge tubes for standby.

*Recommended storage time: at -20°C for up to 3 months.*

14. Y-27632 dihydrochloride (100 mM, 10,000×): 1.56 mL of ultra-pure water supplemented with 50 mg Y-27632. Aliquot 5 µL into 1.5 mL microcentrifuge tubes for standby.

*Recommended storage time: at -20°C for up to 6 months.*

### Media

1. Digestion medium I (5 mg/mL, 1×):

4.85 mL AdDMEM/F12^+++^

50 µL DNase I (10 mg/mL)

100 µL Collagenase type II (250 mg/mL)

0.5 µL Y-27632 dihydrochloride (100 mM)

*Recommended storage time: prepared fresh, and prewarmed in a 37°C water bath before use.*

2. Organoid culture medium (50 mL, 1×):

48.02 mL AdDMEM/F12^+++^

1 mL B-27 supplement (50×)

125 µL N-acetyl-L-cysteine (500 mM)

500 µL Nicotinamide (1 M)

250 µL R-Spondin 1 (100 µg/mL)

50 µL Noggin (100 µg/mL)

25 µL EGF (100 µg/mL)

2.5 µL FGF-7 (100 µg/mL)

5 µL FGF-10 (100 µg/mL)

16.7 µL SB202190 monohydrochloride hydrate (30 mM)

1 µL A83-01 (25 mM)

5 µL Y-27632 dihydrochloride (100 mM)

*Recommended storage time: at 4°C for up to 2 weeks.*

## Methods

### Acquisition and transportation of clinical samples TIMING 30 min

**Figure 1:**
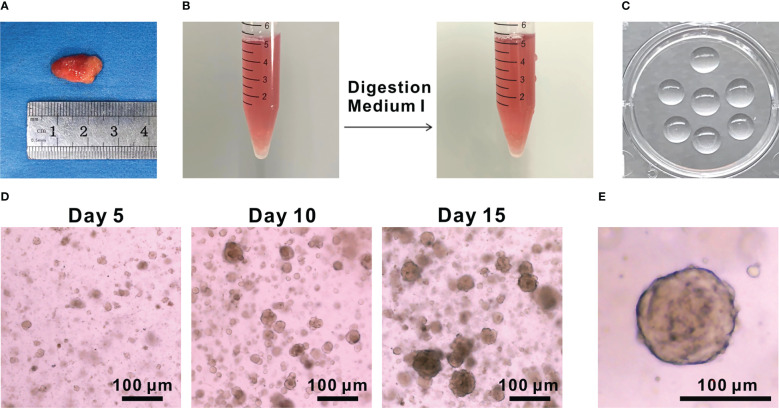
Development of PTC organoids. **(A)** Image of PTC tissue. **(B)** Images of PTC tissues before and after digestion with Digestion Medium (I) **(C)** Image of Matrigel domes plated in a 6-well culture plate. **(D)** Representative bright-field images of PTC-3 organoids at 5, 10 and 15 days, respectively. Scale bar, 100 µm. **(E)** An enlarged image of PTC-3 organoid. Scale bar, 100 µm.

1. Prior to the operation, prepare 10 mL of ice-cold AdDMEM/F12^+++^, ice and an ice box.

2. Collect the fresh tumor tissue sample (recommended sample volume > 5 mm^3^) from surgery (**Figure 1A**), and then put into ice-cold AdDMEM/F12^+++^medium until processing.

**PAUSE POINT:** Fresh tumor tissues can be stored in AdDMEM/F12^+++^ for up to 18 h at 4°C. However, we recommend that tissue samples should be processed as soon as possible to improve the success rate of organoid establishment.

3. This study was approved by the Human Ethics Committee of Peking University Shenzhen Hospital (No. 2022-147), and informed consent from all patients was obtained in accordance with the Declaration of Helsinki.

### Tissue processing and derivation of PTC organoids TIMING 2 h

4. On arrival, wash the tumor tissue 3 times with cold PBS until no significant blood remained, and divide it into several pieces using scissors. Snap frozen 3 pieces and store them at -80°C for DNA/RNA isolation. Fix 1 piece in 10% neutral-buffered formalin for histological analysis. Mince and dissociate the remaining tumor tissues for organoid derivation.

**NOTE:** To reduce contamination of normal thyroid cells, the margins of tumor tissue can be trimmed appropriately.

5. Incubate the minced tissues with 5 mg/mL Digestion Medium I for 40-60 min at 37°C under shaking at 110 rpm (**Figure 1B**). Use 1 mL of Digestion Medium I per 50 mg of tumor tissue. Add AdDMEM/F12^+++^ medium top to 10 mL and then centrifuge at 300 × g for 5 min. Remove the supernatant to obtain cell pellets.

6. Resuspend the cell pellets in 5 mL of AdDMEM/F12^+++^. Filter the mixture through a 70-µm cell strainer to remove chunks of undigested fragments. Centrifuge the tube again at 300 × g for 5 min. Discard the supernatant carefully.

**NOTE:** At the same time, put the 6-well cell culture plate in the incubator to prewarm for later use. Get an ice box ready.

7. Resuspend the cells with 50 µL of cold organoid culture medium and 200 µL of cold Matrigel.

**NOTE:** Do not dilute the Matrigel too much. The final content of Matrigel should not less than 75%.

**NOTE:** Matrigel should be kept in ice to avoid solidification. The tips need to be precooled in cold sterile PBS before touching Matrigel.

8. Mix the suspension gently and slowly on ice to avoid introducing air bubbles.

9. Seed Matrigel-cell mixture as 45 µL of droplets (~15, 000 cells/drop) into one well of a prewarmed 6-well plate. Place the plate right side up for 5 min and upside down for another 15 min in an incubator (5% CO_2_, 37°C) (**Figure 1C**).

**NOTE:** Generally, 5-6 drops are plated into each well of a plate.

**NOTE:** On average, we can obtain ~100,000 cells/mL from 50 mg specimen.

**NOTE:** The Matrigel is beginning to solidify immediately after leaving the ice, so the operation of plating needs to be skillfully and quickly.

**NOTE:** Make sure the droplets are solidified before inverting the cell plate.

10. Add 2.5 mL of organoid culture medium to each well after the Matrigel has solidified completely.

**NOTE:** Add the culture medium gently down the sidewall of each well and completely submerge the Matrigel domes.

**NOTE:** Organoid culture medium should be prewarmed, so as not to liquify the solidified Matrigel back.

### Maintenance and passaging of PTC organoids TIMING 1 h

11. Refresh the medium every 3 days. Representative pictures of PTC organoids under light microscope were shown (**Figures 1D, E** **)**.

**NOTE:** To maximize organoid generation from isolated cells, Y-27632 dihydrochloride should be added during the first 2 weeks of culture. Afterwards, using the organoid culture medium without Y-27632 dihydrochloride.

12. After 2-3 weeks of culture, resuspend the Matrigel domes (5-6 domes per well) from each well of the plate with 2 mL TrypLE Express containing 10 μM Y-27632 dihydrochloride. Incubate the organoids at 37°C for approximately 5 min with a vigorous manual shake per minute.

**NOTE:** We can dissociate organoids into smaller cell pellets by pipetting them up and down with a 3 mL pasteur pipet.

**NOTE:** Generally, PTC organoids are passaged every 2 weeks at a 1:2 to 1:3 dilution. When half of the PTC organoids are larger than 100 µm in diameter or many organoids join together and become overcrowded, passage should be performed.

13. Add 2 mL of AdDMEM/F12^+++^ (containing 10% FBS) to stop digestion. Centrifuge at 300 × g for 5 min, and then remove the supernatant.

14. Wash the cell pellets with another 2 mL of AdDMEM/F12^+++^ to remove TrypLE Express completely. Centrifuge at 300 × g for 5 min and discard the supernatant carefully to collect the cell pellets.

15. Put the tube on ice, and resuspend the cell pellets with 50-100 µL of cold organoid culture medium according to the amount of precipitated cells.

16. Add appropriate amounts of Matrigel (usually 200-300 µL per well) to the tube and mix gently. Continue with the “Step 9”.

**Figure 2:**
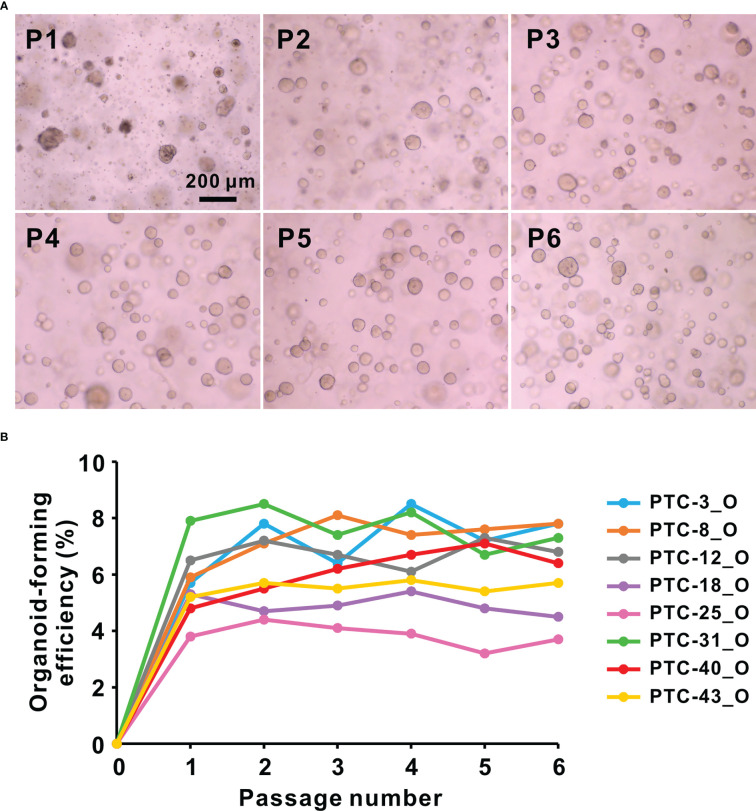
**(A)** Representative bright-field images of PTC-8 organoids at passage (P) 1 to 6. Scale bar, 200 µm. **(B)** Organoid-forming efficiency of 8 representative PTC organoid lines during passaging. O, organoid.

**NOTE:** Cell debris and impurities will be reduced with serial passaging (**Figure 2A**).

### Cryopreservation of PTC organoids TIMING 40 min

17. Collect PTC organoids as mentioned in “Step 12-14”.

18. Count cells with a hemocytometer, and collect ~100,000 cells with 800-1000 µL of Recovery Cell Culture Freezing Medium. Transfer the resuspension solution into a cryogenic vial, and put the vial into a freezing container at -80°C.

19. For long-term preservation, frozen cryogenic vials should be transferred to the liquid nitrogen tank the next day.

**PAUSE POINT:** PTC organoids can be kept for up to 1 month in freezing container at -80°C and in liquid nitrogen for up to 2 years.

### Resuscitation of PTC organoids TIMING 40 min

20. Gently shake the cryogenic vials in a 37°C water bath until completely thawed and transfer the thawed organoids into a 15 mL centrifuge tube.

21. Add 5 mL of prewarmed AdDMEM/F12^+++^ in order to wash the organoids.

22. Centrifuge at 300 × g for 5 min and discard the supernatant carefully.

23. Resuspend the PTC organoids with culture medium and seed them as mentioned in “Step 7-10”.

### Processing of PTC organoids for histology TIMING 2 days

24. Transfer a Matrigel dome from the culture plate into 1mL of 10% neutral formalin buffer in a 1.5 mL tube. Dissociate the Matrigel dome into little fragments by pipetting up and down for 20 times. Fix the organoids at 4°C for 24 h.

**NOTE:** Retain the Matrigel surrounding the organoids, which will keep the organoids’ histological structure more completely.

25. Centrifuge at 1000 × g for 5 min and aspirate the supernatant carefully.

**NOTE:** As 2% agarose is too viscous to resuspend organoids directly, retain a few of formalin, and do not introduce bubbles.

26. Mix 100 mg of agarose with 5 mL of ultra-pure water in a 15 mL tube, and microwave for about 1 min to melt the agarose.

**NOTE:** Before heating, blow the solution evenly with a pipette and heat it until the solution appear to be transparent.

27. Mix the organoids with 50 µL of 2% agarose solution at ~ 50°C.

28. Pipette organoid-agarose mixture as one droplet onto a piece of parafilm. Let the drop solidify for 15 min to form a dome structure, and then invert it for another 15 min to allow the agarose solidified completely.

**NOTE:** The head of P100 tips should be cut off to allow the pipette to mix the agarose solution smoothly.

29. Place the solidified agarose dome into a cassette and perform the following procedures:

**NOTE:** The solidified agarose dome will shrink in volume after dehydration, so it should be wrapped in a piece of filter paper before being placed in a cassette to prevent leakage. This procedure is not required for sufficiently large tissue samples.

**Dehydration:**

1 h in 70% ethanol

1 h in 80% ethanol

1 h in 95% ethanol

45 min in 100% ethanol

**Clearing:**

30 min in 100% ethanol:100% xylene = 1:1

20 min in 100% xylene

20 min in another 100% xylene

**Paraffin infiltration:**

1 h in 100% xylene:100% paraffin = 1:1

1 h in 100% paraffin

1 h in another 100% paraffin

**NOTE:** As a hazardous solvent, xylene is highly volatile. Xylene needs to be operated in the fume hood.

30. Embed samples in paraffin and section at a thickness of 4-µm.

31. To characterize PTC tissues and the derived organoids, hematoxylin and eosin (H&E) staining and immunofluorescence examination should be performed on the paraffin sections.

### H&E staining of PTC organoids TIMING 1.5 h

32. Perform the following procedures:

**Deparaffinization:**

30 min in 100% xylene

30 min in another 100% xylene

**Rehydration:**

5 min in 100% ethanol:100% xylene = 1:1

2 min in 100% ethanol

2 min in another 100% ethanol

2 min in 95% ethanol

2 min in 80% ethanol

2 min in 70% ethanol

2 min in water

**Hematoxylin staining:**

1-3 min in hematoxylin

3 min in water

**Differentiation:**

3 sec in 0.5% hydrochloric acid

2 min in 70% ethanol

2 min in 80% ethanol

2 min in 95% ethanol

**Eosin staining:**

30-40 sec in eosin (95% alcohol)

**Dehydration:**

2 min in 95% ethanol

2 min in another 95% ethanol

2 min in 100% ethanol

2 min in 100% ethanol:100% xylene = 1:1

**Clearing:**

2 min in 100% xylene

2 min in another 100% xylene

**NOTE:** Observe organoid staining under microscope rapidly after hematoxylin or eosin staining to determine whether to extend the staining time.

**Figure 3:**
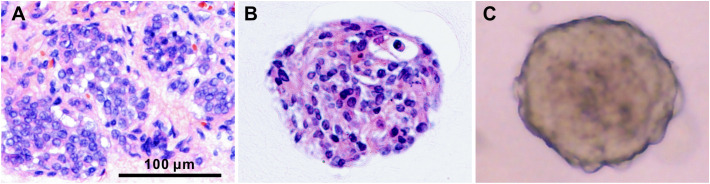
Histological characterization of PTC tissue and the derived organoids. H&E staining of PTC-3 tissue **(A)** and the paired organoid **(B)**, and the bright-field microscopy image of organoid **(C)**. Scale bar, 100 µm.

33. Add neutral resin on the slides and mount with coverslips.

34. Capture the images of stained PTC organoids using a light microscopy. We found that the organoids retained the histological properties of the parental tumors (**Figure 3**).

### Immunofluorescence staining of PTC organoids TIMING 1-2 days

**NOTE:** Dry the slides for 1 hour before the experiment to prevent detachment of tissue/organoid pieces.

35. **Deparaffinization** and **Rehydration** as mentioned in “Step 32”.

36. **Antigen retrieval:**

5 min in PBS.

20 min at 95°C in boiled citrate-EDTA buffer (0.01 M, pH 6.0).

**Blocking:**

Allow the slides naturally cool down to ~ 23°C.

5 min in PBS, and then blot the PBS around the sections.

20 min in ready-to-use normal goat serum, and then blot the blocking solution.

**Incubation with primary antibodies:**

2-3 h at ~ 23°C in primary antibodies at desired dilution.

**PAUSE POINT:** The sections can incubate with primary antibodies to the next day at 4°C.

**Incubation with secondary antibodies:**

3 × 5 min in PBS, and then blot the PBS around the sections.

1 h at ~ 23°C in the dark in Cy3-labeled goat anti-rabbit IgG (H+L) or Cy3-labeled goat anti-mouse IgG (H+L) at 1:300 dilution.

**NOTE:** To stain nucleus, incubate the sections for 15 min at ~ 23°C with DAPI solution (1:1000 dilution).

3 × 5 min in PBS.

37. Add the antifade polyvinylpyrrolidone mounting medium on the slides and mount with coverslips.

**NOTE:** The sections should be stored in a dark environment at 4°C before imaging.

**Figure 4:**
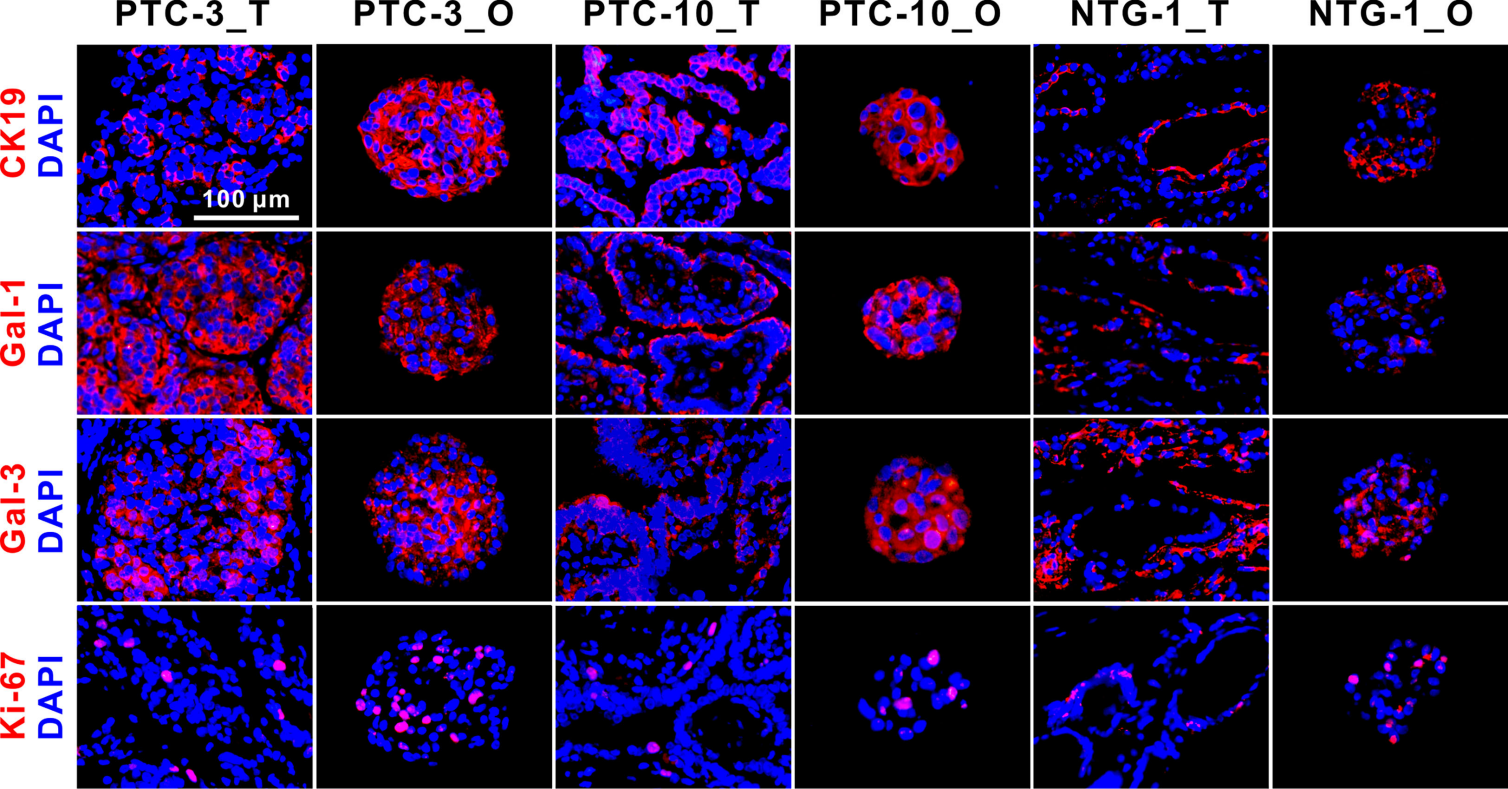
Immunofluorescence staining of CK19, Gal-1, Gal-3, and Ki-67 on PTC- and NTG-derived organoids and the matched tumor tissues. Nuclei were counterstained with DAPI (blue). Passage numbers of these organoid lines were: PTC-3_O, P3; PTC-10_O, P5; NTG-1_O, P4. CK19, Cytokeratin 19; Gal-1, Galectin-1; Gal-3, Galectin-3; NTG, nodular thyroid goiter; T, tumor; O, organoid. Scale bar, 100 µm.

38. Capture the images of stained PTC organoids using a confocal microscopy. We found that the PTC organoids retained the expression profiles of these biomarkers, CK19, Gal-1, Gal-3 and Ki-67 (**Figure 4**).

### RNA isolation and qPCR analysis of PTC organoids TIMING 1 days

39. PTC organoids from different passages can be obtained as “Step 12-14”.

40. Total RNA of organoids is extracted using TRIzol reagent according to the manufacturer’s protocol.

41. The quantity of RNA can be assessed using the NanoDrop 1000 Spectrometer, and the integrity of RNA can be checked with 1.5% agarose gel running.

**PAUSE POINT:** RNA samples can be stored at -80°C for up to 1 year. Avoid repeated freeze and thaw.

**NOTE:** Nuclease-free distilled water should be applied in RNA extraction.

42. RNA samples can be employed to quantify gene expression levels in organoids by qPCR analysis. Approximately 1 μg of RNA is first treated with DNase I and reverse transcribed into cDNA using the RevertAid First Strand cDNA Synthesis Kit with random hexamers following the manufacturer’s instruction.

**PAUSE POINT:** The cDNA samples can be stored at -80°C for years.

43. Prepare a stock solution of both forward and reverse primers in ultra-pure PCR grade water at a final concentration of 10 μM.

44. The qPCR reaction is performed in a total volume of 10 μL, containing 1 μL of cDNA template, 0.3 μM of each primer, and 5 μL of Bestar^®^ Sybr Green qPCR Master Mix.

**NOTE:** The reaction should be run in triplicate per sample. No reverse transcriptase and no cDNA template controls should be included in each assay.

45. Keep the 384-well plate cold with a plate cooler and add 9 μL of the premixture followed by 1 μL of the cDNA template to each well.

46. Seal the plate with a clean adhesive strip, and then centrifuge at 1,000 × g for 1 min.

47. Perform qPCR on a Roche LightCycler 480 detection system. The cycling parameters are 95°C for 5 min; 40 cycles of 95°C for 15 s, 58°C for 1 min, and 72°C for 20 s. The specificity of qPCR amplification can be confirmed by melt-curve analysis (5 s at 95°C, 1 min at 65°C, and 20 s at 95°C), gel electrophoresis, and sequencing of PCR products.

**NOTE:** The qPCR protocol is designed for a 10 uL reaction mixture in the 384-well plate and for the use of Bestar^®^ Sybr Green qPCR Master Mix (DBI^®^ Bioscience) and a LightCycler 480 detection system (Roche). For different qPCR program, please refer to the manufacturer’s guidelines of your reagents and machine of choice. However, the general design principles are similar as described here.

**NOTE:** A melting curve analysis is essential to confirm that only a single specific product is formed. If two or more peaks are obtained, the reaction should be performed again or the primers should be redesigned.

48. Export the data and calculate the average of relative concentration of DNA for each sample from 3 or more replicates. The target gene expression levels are presented as the copy number ratios to the reference gene.

## Results and discussion

### Establishment of patient-derived PTC organoids

**Figure 5:**
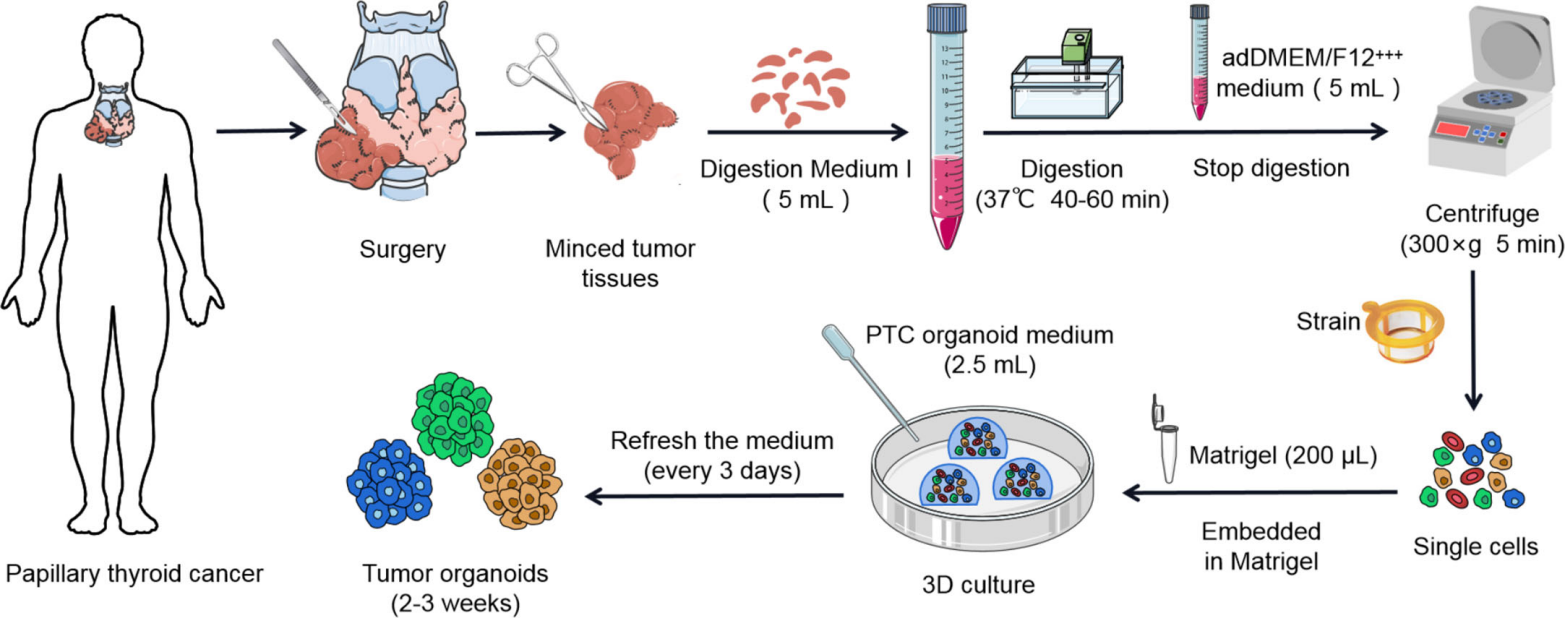
Flow diagram of establishment of patient-derived PTC organoid lines.

Here, we present a simple and effective way to generate PTC organoids. Freshly resected PTC tissues were collected from clinical surgery. Clinical characteristics of patients were described in **Supplementary Table 1**. PTC tissues were digested into single-cell populations through mechanical disruption and enzymatic digestion, embedded into Matrigel and finally covered with the organoid culture medium (**Figures 5**, **1A-C**). Several other groups have previously reported similar thyroid cancer organoids derived from human tumor tissues or cell lines (21–23). PTC organoids usually formed within 1 week after plating (**Figure 1D**). The majority of the PTC organoids displayed compact and solid structures (**Figures 1D, E** **)**. By observing the growth process of PTC organoids, we found that most of them were monoclonal growth, which provides good materials for the study of tumor heterogeneity.

Usually, PTC organoids should be passaged after 2-3 weeks of culture at a 1:2 to 1:3 dilution. These PTC organoid cultures have been propagated through serial passages, as well as successfully cryopreserved and resuscitated (**Figure 2**). Organoid lines successfully cultivated over 3 passages were regarded as success in organoid establishment. Using this fully detailed method, organoid lines can be established from human thyroid cancer samples with a success rate of 77.6% (38/49). One of the possible reasons for failed cases in organoid establishment may be the small size of initial specimen. The organoid-forming efficiency of 8 representative PTC organoid lines during passaging was examined, by referring to the methods previously used (24, 25). This procedure has repeated over 6 passages for these PTC organoids, with the organoid-forming efficiencies between 3.3% and 8.5% of different lines (**Figure 2B**), suggesting the potential self-renewal ability of PTC organoid-derived cells.

### PTC organoids retain the histopathological characteristics and gene expressions of original tumors

To identify whether PTC organoids recapitulate the pathological and morphological features of the parental tumors, we performed H&E and immunofluorescence staining of tumor tissues and the corresponding organoids. As shown in **Figure 3**, PTC organoids were similar to the original tumor tissue in morphological and cytological characteristics, including eosinophilic or clear cytoplasm, large overlapping nuclei, irregular nuclear contour, and pale or ground-glass nuclear chromatin, in accordance with what have been reported previously (2, 26, 27).

Some specific molecular markers were selected to identify PTC tissues and the matched organoids (**Figure 4**). CK19, Gal-1 and Gal-3 are commonly used markers for differentiated thyroid cancer, and Ki-67 is a cell proliferation marker. Immunofluorescence staining showed that CK19, Gal-1, Gal-3 and Ki-67 were expressed in both PTC and nodular thyroid goiter (NTG) tissue-organoid pairs. CK19 and Gal-1 were shown to be highly expressed in PTC, but weakly expressed in NTG tissues and the derived organoids. The expression patterns of these makers in PTC- and NTG-derived organoids were consistent with the corresponding tumor tissues. Taken together, these results suggest that PTC organoids are able to maintain the morphological and pathological features of tumor tissues from which they are derived.

**Figure 6:**
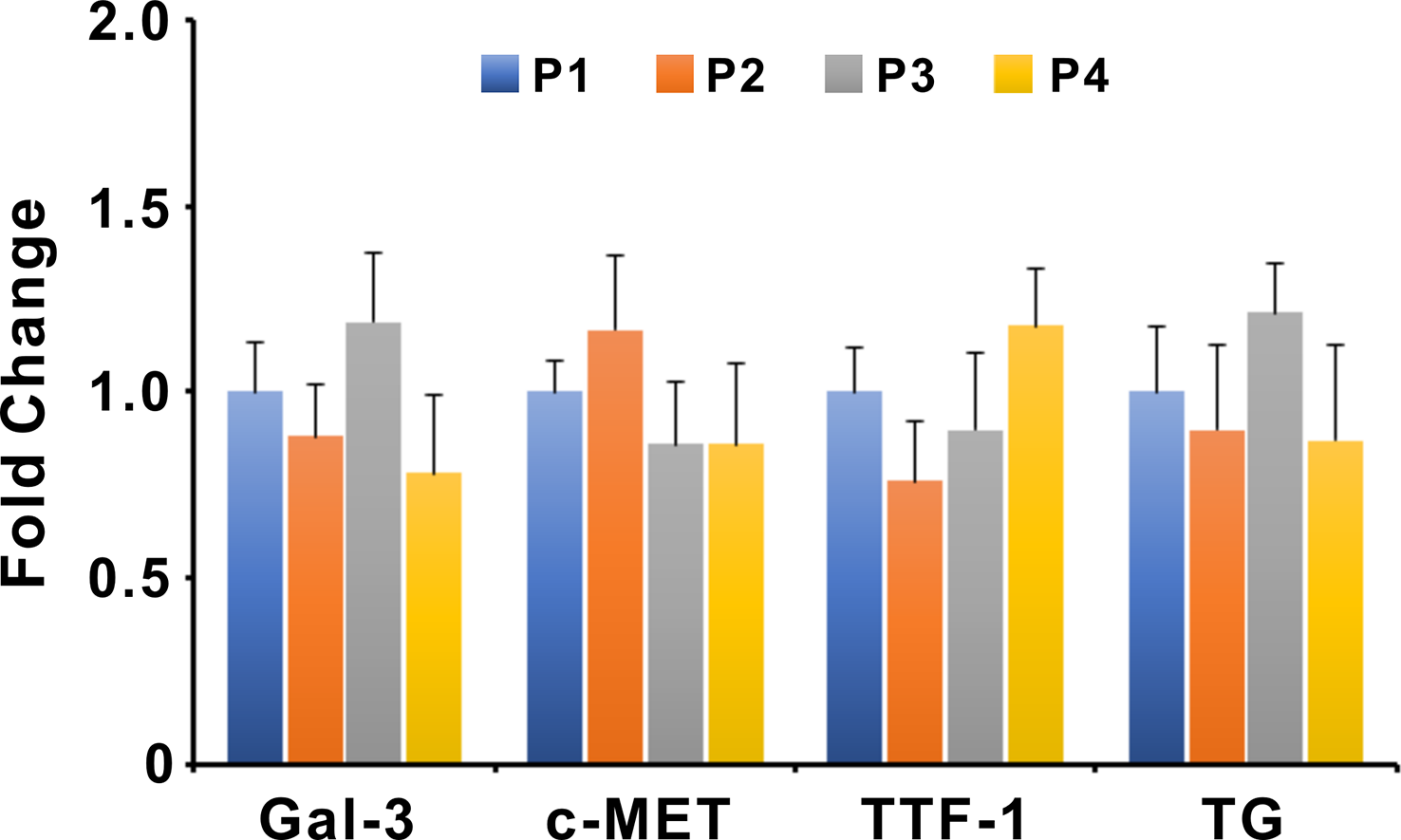
Expression of Gal-3, c-MET, TTF-1, and TG in different passages of PTC organoids. Bars represent mean ± SEM of 5 organoid lines, normalized to passage 1. Gal-3, Galectin-3; TG, Thyroglobulin.

The gene expression levels of Gal-3, c-MET, TTF-1, and Thyroglobulin (TG) in organoids of different passages were examined by qPCR (**Figure 6**). Expression levels of target genes were normalized to the reference gene GAPDH. The nucleotide sequences of primer pairs were listed in **Supplementary Table 2**. The qPCR results showed that the expression levels of Gal-3, c-MET, TTF-1, and TG were not significantly altered between different passages of PTC organoids, suggesting the stable expression of specific genes during passaging.

## Troubleshooting

### Problem 1. Low cell yield after tissue digestion

Calcification is usually present in PTC tissues. The higher degree of calcification, the smaller amount of cells may be available. Moreover, Calcified tissues are difficult to mince and digest sufficiently. For calcified specimens, we advise to incubate them up to 1 hour with Digestion medium I. Meanwhile, gently pipette up and down 15-20 times using 5 mL pasteur pipet every 10 min to promote the digestion efficacy.

### Problem 2. Small number of organoids were obtained after passaging

Compared to several cancer organoids (e.g., breast cancer organoids), thyroid cancer organoids grow more slowly. We can appropriately delay the passage time according to the growth rate and diameter size of organoids. More small-diameter organoids can be obtained by rapid blowing up and down with a pipette when the diameter of organoids increases above 100 µm.

### Problem 3. Dying organoids

The culture of organoids requires good cell viability. We recommend that the derivation of PTC organoids should be performed as soon as possible when tumor samples were obtained from the operating room. In addition, the Rho kinase inhibitor Y-27632 should be added during the dissociation and cryopreservation steps, as well as the first 2 weeks of culture to avoid detach-induced anoikis.

## Data availability statement

The original contributions presented in the study are included in the article/**Supplementary Material**. Further inquiries can be directed to the corresponding authors.

## Ethics statement

The studies involving human participants were reviewed and approved by the Human Ethical Committee of Peking University Shenzhen Hospital (approval No. 2022-147). The patients/participants provided their written informed consent to participate in this study.

## Author contributions

HY, QL, JZ and DC contributed original experimental data. HY, DC, and YZ wrote the manuscript. All authors agree to be accountable for the content of the work. All authors contributed to the article and approved the submitted version.
