# Supplementary material for: Establishment of papillary thyroid cancer organoid lines from clinical specimens
Source: Front Endocrinol (Lausanne). 2023 Mar 13;14:1140888. doi: 10.3389/fendo.2023.1140888 (PMC10040568; doi:10.3389/fendo.2023.1140888)
Supplement: Supplementary file 2 [file Table_2.doc]

**Supplementary Table 2.** Sequences of oligonucleotide primers used in qPCR.

| **Primer name** | **Sequence (5’-3’)** |
| --- | --- |
| Gal-3-F  Gal-3-R  c-MET-F  c-MET-R  TTF-1-F  TTF-1-R  TG-F  TG-R  GAPDH-F  GAPDH-R | ATGCAAACAGAATTGCTTTAGATT  AGTTTGCTGATTTCATTGAGTTTT  TGGGAAGAAGATCACGAAG  TGTAGATTGCAGGCAGACAGA  CGCGTTTAGACCAAGGAAC  GAGTGTGCCCAGAGTGAAG  ATGCACTGGGGAACTCAAAG  TGAGGCTGAGAACACAATGG  CCATGGAGAAGGCTGGGG  CAAAGTTGTCATGGATGACC |

F: Forward primer; R: Reverse primer; Gal-3: Galectin-3; TG: Thyroglobulin.
